# Overrunning in clinical trials: some thoughts from a methodological review

**DOI:** 10.1186/s13063-020-04526-5

**Published:** 2020-07-21

**Authors:** Ileana Baldi, Danila Azzolina, Nicola Soriani, Beatrice Barbetta, Paola Vaghi, Giampaolo Giacovelli, Paola Berchialla, Dario Gregori

**Affiliations:** 1 Unit of Biostatistics, Epidemiology and Public Health, Department of Cardiac, Thoracic and Vascular Sciences, University of Padua, Via Loredan 18, 35121 Padova, Italy; 2 Department of Translational Medicine, University of Eastern Piedmont, 28100, Novara, Italy; 3 Department of Biostatistics, Rottapharm Biotech, 20900, Monza, Italy; 4 Department of Clinical and Biological Sciences, University of Torino, 10100, Turin, Italy

**Keywords:** Overrunning, Deletion method, Combining *p* values, Repeated confidence interval

## Abstract

**Background:**

In sequential and adaptive trials, the delay that happens after the trial is stopped, by a predetermined stopping criterion, takes the name of overrunning. Overrunning consists of extra data, collected by investigators while awaiting results of the interim analysis (IA). The inclusion of such extra data in the analyses is scientifically appropriate and follows regulatory advice. Nevertheless, its effect from a broader perspective is unclear.

**Methods:**

This article aims at clarifying the overall impact of including such overrunning data, providing first a revision, and then a comparison of the several approaches proposed in the literature for treating such data. A simulation study is performed based on two real-life examples.

**Results:**

The paper shows that overrunning inclusion could seriously change the decision of an early conclusion of the study. It also shows that some of the methods proposed in the literature to include overrunning data are more conservative than others.

**Conclusion:**

The choice of a more or a less conservative method could be considered more appropriate depending on the endpoint type or the design type.

## Background

Increasingly, sequential procedures are being implemented in modern clinical trials [1–3]. The methodology for conducting a trial sequentially has been extensively developed, evaluated, and documented. Error rates can be accurately preserved, and valid inferences are drawn. Group sequential designs (GSDs) have become a frequently used approach for interim monitoring in clinical trials stemming from several research settings [4]. GSDs control the overall type I error at a pre-specified level through an alpha spending function, which provides a specific statistical stopping criterion (or boundary) for each interim analysis (IA) [5–7]. Such considerations are at the bottom line of adaptive designs as well [8].

The GSD is a flexible strategy for planning a clinical trial without compromising the study’s validity or integrity [9]. For example, the GSD interim trial monitoring procedure leads to minimizing the exposure to the potential toxicity effect of a new treatment or to minimize the trial duration once it has been evidenced a strong risk or benefit for the patient [10]. Moreover, the flexible GSD would allow for an interim sample size reassessment to accommodate for an eventually smaller than expected by the study design effect size [11].

In a GSD, IA serves the primary purpose of terminating the trial when futility or superiority of one of the interventions becomes clear, according to pre-specified stopping rules. Also, they allow checking for adverse side effects, accrual, compliance, contamination, and protocol violations.

Early trial termination has understandable economic motivations (resource-saving) as well as ethical reasons, such as allowing patients to benefit from a useful treatment before the end of the study or reducing their exposure to unnecessary risks.

Practically, it commonly happens in many sequential trials that data continue to be also collected if a stopping criterion has been reached [12]. This phenomenon is referred to as overrunning. The main cause of overrunning is the time delay between the recruitment of the subject and the actual observation and evaluation of the primary outcome. Another common cause of overrunning may be the decision (e.g., by the sponsor who financially supports the study) to continue the trial even if a stopping criterion has been reached [13]. This choice, if not in conflict with ethical reasoning, could be made mainly to create a robust safety database, containing data from more patients, as an aid to support the efficacy results as derived from the interim analysis. According to regulatory bodies, overrunning data collected by the trial protocol are considered valid and should be included in the analyses [3, 14–16]. Nevertheless, they have the potential to influence the results and to change the conclusions of the trial as derived from the IA.

Over the years, many statistical approaches to dealing with overrunning have been proposed. The deletion method [12] includes overrunning data, ignoring the IA that has led to the stopping of the trial. The methods that combine *p* values [17] are based on the combination of two different analyses, one carried out on the sequential portion of the data and the other one on the overrunning part, by weighting their *p* values with fixed or random weights.

The repeated confidence interval (RCI) method [18] is a further, flexible, and appealing alternative to adopt for the overrunning problem. This method does not need adjustments for overrunning, but it pays this flexibility regarding the conservation of the quoted interval [12]. The effect of the RCI method used to deal with overrun data is still poorly addressed.

The current literature [19] evaluates the effect on the trial conclusion of the different statistical methods used to handle with overrunning data analysis without considering differences in study design purposes (non-inferiority, superiority, etc.).

The primary aim of this paper is to understand the effects of including overrunning data on non-inferiority or superiority trial conclusions.

This work also aimed to compare behaviors of existing methods investigating whether and how the size of the overrunning data affects the type I error and in analyzing the potentialities of the examined methods. Furthermore, we want to evaluate the strength of the IA conclusions if overrunning data occur. For these purposes, simulation studies are performed using two example trials and considering multiple scenarios.

The first example trial is based on a superiority trial based on the ASCLEPIOS study [20], with a slightly modified setting concerning the original one presented in Sooriyarachchi et al. [19], while the second example trial refers to a multicenter phase III trial for non-inferiority of a test drug compared to a reference drug (labels have been hidden for confidentiality reasons).

## Methods

### Group sequential design

In the classical two-arm parallel trial, the subjects are randomly assigned to an experimental (*E*) and a control (*C*) treatment group. The advantage on a major endpoint between *E* and *C* is expressed by a parameter *θ*, and the sample size is chosen, for an expected clinical effect, fixing a priori the type I (*α*) and type II (*β*) error probabilities. Type I error is the probability to declare positively for an experimental effect, when this effect is absent (*θ* = 0), while type II error is the probability to exclude an experimental effect, when in reality the effect exists (*θ* ≠ 0). The power is given by 1 − *β*.

The sample size is chosen to achieve expected clinical results, fixing the error probability rates according to a GSD in which *K* IAs are planned. At the *k* − *th* IA, the response on the primary endpoint is summarized by the pair of statistics (*Z*_*k*_, *V*_*k*_). Here, *Z*_*k*_ represents the efficient score statistics for the superiority of *E* in respect to *C*, and *V*_*k*_ denotes Fisher’s observed information. It is assumed that conditional to *V*_*k*_*, Z*_*k*_ is a normal random variable:

1 $$ {Z}_k\dot{\sim}N\left(\theta {V}_k,{V}_k\right). $$

Conditional on the sequence {*V*_1_, *V*_2_, …}, *Z*_1_ and the increments *Z*_*k*_ − *Z*_*k* − 1_ (*k* = 2, 3, …) are independent. Alternatively, the definition corresponds to assuming that conditional on the sequence {*V*_1_, *V*_2_, …}, the joint distribution of {*Z*_1_, *Z*_2_, …} is that of a standard Brownian motion with drift *θ* observed at the time {*V*_1_, *V*_2_, …}.

Stopping criterion is determined by group sequential test [5, 6], where the sequence of *p* values for (*Z*_1_, *Z*_2_, …*Z*_*K*_), computed recurring by the Fairbanks and Madsen ordering [21] and according to the trial design, is compared concerning an opportune sequence (*α*_1_, *α*_2_, …, *α*_*K*_) of significance levels, chosen to control the type I error probability. The trial is not stopped until the null hypothesis continues not to be rejected. The *p* values represent the probability of observing a value more extreme than *Z*_*k*_ in the *k* − *th* IA conditionally to have not stopped the trial at the previous IAs.

A GSD can be summarized by three main quantities: the nominal significance levels (*α*_*k*_), the error spent (*π*_*k*_), and the achieved levels in power (1 − *β*_*k*_). The nominal significance level (*α*_*k*_) represents the threshold for the *p* value to declare the study conclusion. The error spent (*π*_*k*_) is the theoretical conditional probability of stopping at stage *k* and rejecting the null hypothesis when it is true it holds that *π*_1_ + … + *π*_*K*_ = *α*. The power achieved (1 − *β*_*k*_), such that $\sum \limits_{k=1}^K\left(1-{\beta}_k\right)=1-\beta$, is the probability that the trial stops at the *k* − *th* stage and so to reject the null hypothesis when it is false.

### Overrunning methods

The methods to incorporate overrunning data in the analysis are, often, direct extensions of methods of analyzing data from a sequential trial without overrunning. The notation used to outline these methods is taken from Whitehead [22] and Jennison and Turnbull [18, 23].

When overrunning occurs after the trial is stopped at the *k* − *th* IA, then the analysis that includes the overrunning data could be considered as an unplanned (*k* + 1) − *th* IA, so it will have associated the statistics *Z*_*k* + 1_ and *V*_*k* + 1_. The contribution of the overrunning data to the analysis could be determined by the quantities *Z*_*O*_ = *Z*_*k* + 1_ − *Z*_*k*_ and *V*_*O*_ = *V*_*k* + 1_ − *V*_*k*_.

#### Deletion method

The deletion method corresponds to the approach introduced by Whitehead [12]. When this method is applied, the IA that led to the fulfillment stopping criterion is deleted and replaced with a new one that includes the overrunning data. This means that if the trial reached the stopping criterion at the *k* − *th* IA, then deletion method reduces to recompute the *p* value, on the statistics (*Z*_*k* + 1_, *V*_*k* + 1_) instead of on the statistics (*Z*_*k*_, *V*_*k*_).

#### Combining *p* values

The method of combining *p* values [17] distinguishes and analyzes the sequential (until recruitment termination) and the overrunning portions of the trial separately.

These analyses are then combined as:

2 $$ P\left(\theta \right)=1-\Phi \left[{w}_1\times {\Phi}^{-1}\left\{{P}_T\left(\theta \right)\right\}+{w}_2\times {\Phi}^{-1}\left\{{P}_O\left(\theta \right)\right\}\right], $$

*P*_*T*_(*θ*) and *P*_*O*_(*θ*) are the *p* values computed respectively for the sequential and the overrunning data.

Theoretically, the method makes it possible to combine the sequential and the overrunning portions for any pair of weights such that ${w}_1^2+{w}_2^2=1$. Let *V*_*K*_ be the information at the observed stopping time *k* and *V*_*O*_ be the overrun information. The *random weights* may be defined as:

3 $$ {w}_1=\sqrt[]{\frac{V_K}{V_K+{V}_O}}\ \mathrm{and}\ {w}_2=\sqrt[]{\frac{V_O}{V_K+{V}_O}}, $$

The weights are related to Fisher’s information in the two portions of data. Another choice could be the so-called fixed weights [19]:

4 $$ {w}_1^{\prime }=\sqrt{\frac{E\left[{n}_K;{H}_0\right]}{E\left[{n}_K;{H}_0\right]+E\left[{n}_O;{H}_0\right]}}\ \mathrm{and}\ {w}_2^{\prime }=\sqrt{\frac{E\left[{n}_O;{H}_0\right]}{E\left[{n}_T;{H}_0\right]+E\left[{n}_O;{H}_0\right]}}. $$

For this option, the weights are related to the expected value under the null hypothesis of the sample sizes in the sequential and overrunning portions of the trial. This choice of weights required [19] that the value of the expected overrunning size should be expressed a priori, in the trial design description.

#### Repeated confidence interval

The repeated confidence interval (RCI) method, for a trial with *K* IAs, leads to a 1 − *α* level sequence of confidence intervals {*I*_*k*_ : *k* = 1, …, *K*} for the parameter *θ*, where each *I*_*k*_ is built from the information available at analysis *k* and

$$ {P}_{\theta}\left\{\theta \in {I}_k:k=1,\dots, K\right\}=1-\alpha . $$

In the method developed by Jennison and Turnbull [18], the repeated confidence intervals are obtained by inverting a family of group sequential tests [5, 6]. A two-sided group sequential test of the null hypothesis *H*_0_ : *θ* = *θ*_0_ with *K* IAs and type I error probability *α* rejects *H*_0_ at the *k* − *th* analysis if |*Z*_*k*_ − *θ*_0_*V*_*k*_| ≥ *c*_*k*_(*V*_*k*_), for *k* = 1, …, *K*. The critical values *c*_*k*_(*V*_*k*_) depend on the form of the test used. For the O’Brien and Fleming design [6, 18, 23], the critical values are set equal to ${c}_k\left({V}_k\right)={C}_B\left(K,\alpha \right)\sqrt{V_k/k}$.

The *k* − *th* repeated confidence interval is the set:

5 $$ {\displaystyle \begin{array}{ccc}{I}_k& =& \left\{{\theta}_0:|{Z}_k-{\theta}_0{V}_k|<{c}_k\left({V}_k\right)\right\}\\ {}& =& \left(\frac{Z_k-{c}_k\left({V}_k\right)}{V_k};\frac{Z_k+{c}_k\left({V}_k\right)}{V_k}\right)\end{array}} $$

of values of *θ*_0_ accepted by the specific tests. The stopping criterion for the RCI method is to stop the study at the *k* − *th* analysis if *θ*_0_ ∉ *I*_*k*_(*θ*).

The main advantage of the RCI method is that it does not need an adjustment for overrunning. If the study is stopped at the *k* − *th* analysis and overrunning occurs, then the repeated confidence interval (*I*_*k*_) is recomputed considering also the overrunning portion of the data. From this point of view, the RCI method is similar to the deletion method, but the *p* values obtained by inverting the confidence intervals of the RCI method differ by construction, from the *p* values obtained with deletion method by the Fairbanks and Madsen ordering [21].

### Example trials

#### ASCLEPIOS superiority trial

The first simulation study is inspired by the ASCLEPIOS study [20]. It tests the superiority of an experimental calcium channel blocker over a placebo control in the immediate treatment of patients experiencing an acute ischemic stroke. The major outcome variable considered in that study was the assessment of the patient Barthel index [24] (plus a state for patient death) 90 days after randomization. Patient recruitment started in October 1989 and stopped in September 1990 after the first IA, based on 140 patients who declared the study drug was ineffective.

A final analysis based on 229 patients, including the patients recruited during the 90 days preceding the trial termination, was conducted in October 1991. The patient responses were not affected by trial termination because when recruitment was stopped, most of the patients had already received their medication. This preserved the validity of the overrunning data.

The setting considered in our simulation study is slightly different from the original ASCLEPIOS study. We took the 90-day mortality rate as the primary endpoint. The trial is thus designed assuming a reduction in the 90-day death rate from 15% in the control arm to 9% in the experimental treatment arm, corresponding to a log-odds ratio of *θ* = 0.58 demonstrated by the two-sided 0.05 level.

According to the O’Brien and Fleming sequential test with three equally spaced IAs, a sample size of 1248 subjects (416 for each IA and 624 for treatment arm) is needed to ensure a power of 0.9, given a two-sided 0.05 level.

Let *n*_*C*_ and *n*_*E*_ be the number of responses and *S*_*C*_ and *S*_*E*_ the number of successes, for the groups *C* and *E*, respectively. Set *n* = *n*_*C*_ + *n*_*E*_, *S* = *S*_*C*_ + *S*_*E*_, and *F* = *n* − *S*. Then, the IAs are based on the statistics *Z* = (*n*_*C*_*S*_*E*_ − *n*_*E*_*S*_*C*_)/*n* and *V* = *n*_*C*_*n*_*E*_*SF*/*n*^3^.

#### Non-inferiority trial

The second simulation study is based on a multicenter phase III trial. The trial, undisclosed for this study, was designed as a double-blind, randomized, parallel-group study for the non-inferiority of a test drug compared to a control drug. The target was to increase the success rate from 45% in the control to 50% in the test drug, with a non-inferiority margin of 15%, which corresponds to a log-odds ratio *θ* =  − 0.20. The power was set to 0.80 to detect *θ* =  − 0.20 as significant at the one-sided 2.5% level. The non-inferiority of the test drug emerged at the first of three IAs. However, the majority of the pre-planned patients had already been recruited so the trial sponsor decided to analyze the full sample to confirm the non-inferiority hypothesis.

The sample size of 198 (99 patients in each treatment group and 66 patients for each IA) was obtained by O’Brien and Fleming’s design.

As in the previous simulation study, the analyses are based on the statistics *Z* = (*n*_*C*_*S*_*E*_ − *n*_*E*_*S*_*C*_)/*n* and *V* = *n*_*C*_*n*_*E*_*SF*/*n*^3^.

Trials are simulated under a null (*H*_0_ : *θ* = 0.65) and an alternative (*H*_1_ : *θ* =  − 0.20) hypothesis. The value *θ* = 0.65 is obtained by assuming a success rate of 45% for the control and 30% for the test drugs, and it represents the more extreme condition in which the non-inferiority of the test drug is rejected.

### Simulation studies

The example trial of the previous section was used to perform two complementary but different sets of simulation studies, in which the behaviors of the overrunning data methods were compared:

1. *First set*. The aim of the first set simulation studies was to study the probabilities of confirming study conclusions. It examined the effect of the overrunning data size on a trial stopped (*i*) at the first or (*ii*) at the second IAs, with *p* values that are close to the O’Brien and Fleming GSD [7] stopping criteria. Both studies consider the effect under both the null (thus stopping the trial was wrong) and the alternative (thus stopping the trial was right) hypotheses of the example trial. Then, studying the changes to these probabilities in confirming the study conclusions is basically like studying the behavior of the methods regarding type I error (under the null hypothesis) and power (under the alternative). The details of the simulation plan were as follows:
  One hundred thousand trials were simulated for each case.
  A trial simulation procedure has been performed considering a simulation and a sequential part:
    - *Simulation part.* The part of the trials influenced by the simulation study was the overrunning part. In both the simulation studies, it was assumed that the expected overrunning size, required for calculating the fixed weights of formula (4), would amount to 10% of the IA sample sizes. This choice is subjective, and for this reason, the same simulated trials were also used to evaluate the sensitivity of the fixed weight method (4), considering several values for the expected overrunning size.
    - *Sequential part.* This part of the trial contains the minimum number of successes (for the experimental arms) to cross the O’Brien and Fleming stopping criterion, so it is common in all of the simulated trials considered.

2. *Second set.* This set of simulation studies had the goal to examine the robustness of the methods: to understand how strong should be the evidence for the alternative hypothesis to ensure, in the presence of overrunning data, a high probability of confirming the study conclusions.
  Again, these evaluations were done for the first and the second IAs. Similarly to what was done for the first set of studies, the simulated trials are composed of two parts: the sequential (until the IA considered) and the overrunning part. The overrunning part is the same used for the first set of simulation studies. This set of studies could be seen as the replication of the first set many times, but increasing the evidence in favor of the alternative hypothesis. More evidence for the alternative hypothesis was obtained by adding successes for the experimental arm in the sequential part of the trial.

For this set of studies, only the simulated trials under the alternative hypothesis were considered, to focus on the behaviors of the various methods in the most favorable scenario (the IAs reject the null hypothesis, and the overrunning data are on the support of the alternative hypothesis).

In both the simulation study sets, the overrunning data sizes were always increased by 1%, and the confirmation rates are ever given by the ratio of the simulated trial that continues to support the trial termination.

The *p* values of the overrunning method were compared with nominal significance levels adapted by Wang and Tsiatis [25] at the portion of the trial observed, thus including the overrunning parts.

## Results

**Fig. 1:**
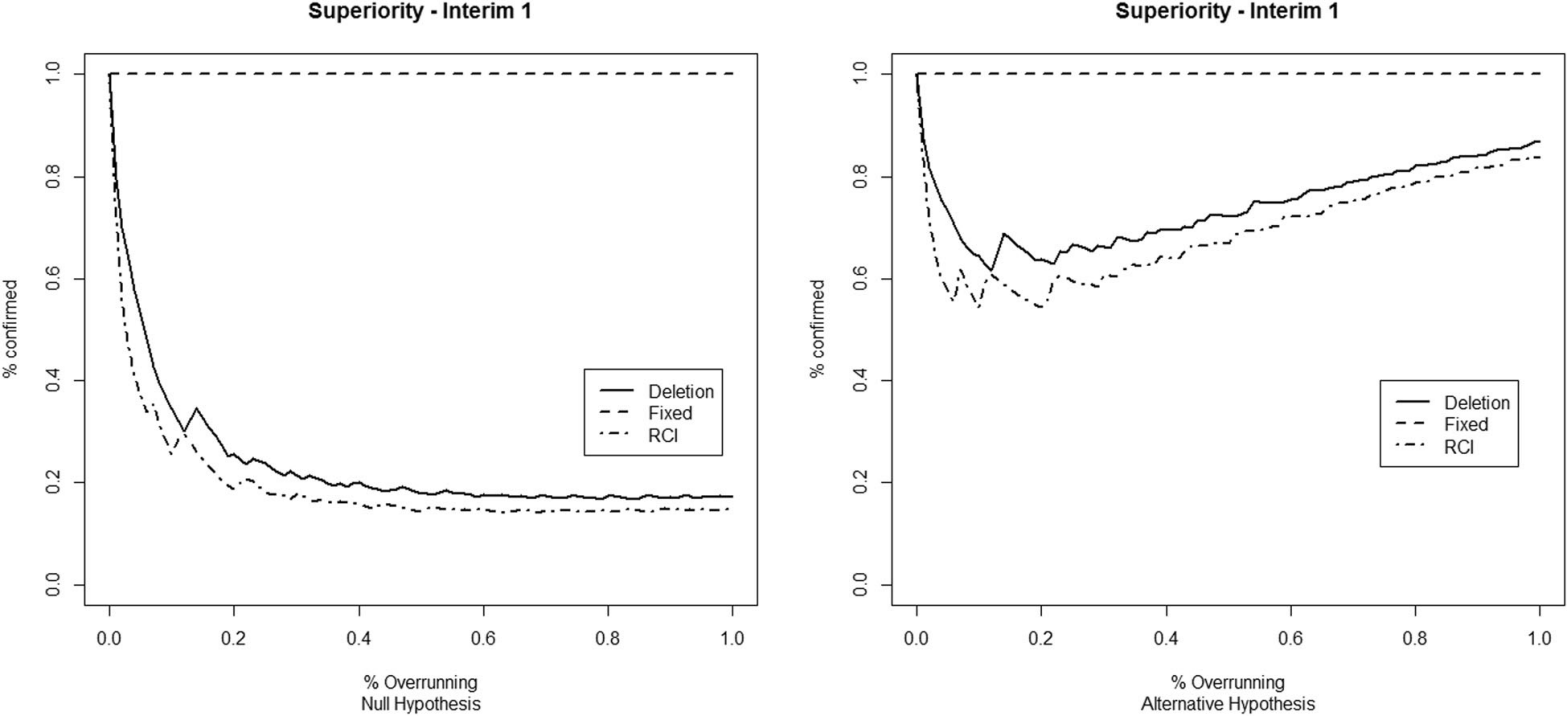
Percentage of confirming the study conclusions by the different overrunning methods at the first interim analysis. The considered trial refers to the minimum number of successes to obtain the *p* value that crosses the O’Brien-Fleming stopping bound. Data simulated under the null (left) and alternative (right) hypotheses of the superiority example

**Fig. 2:**
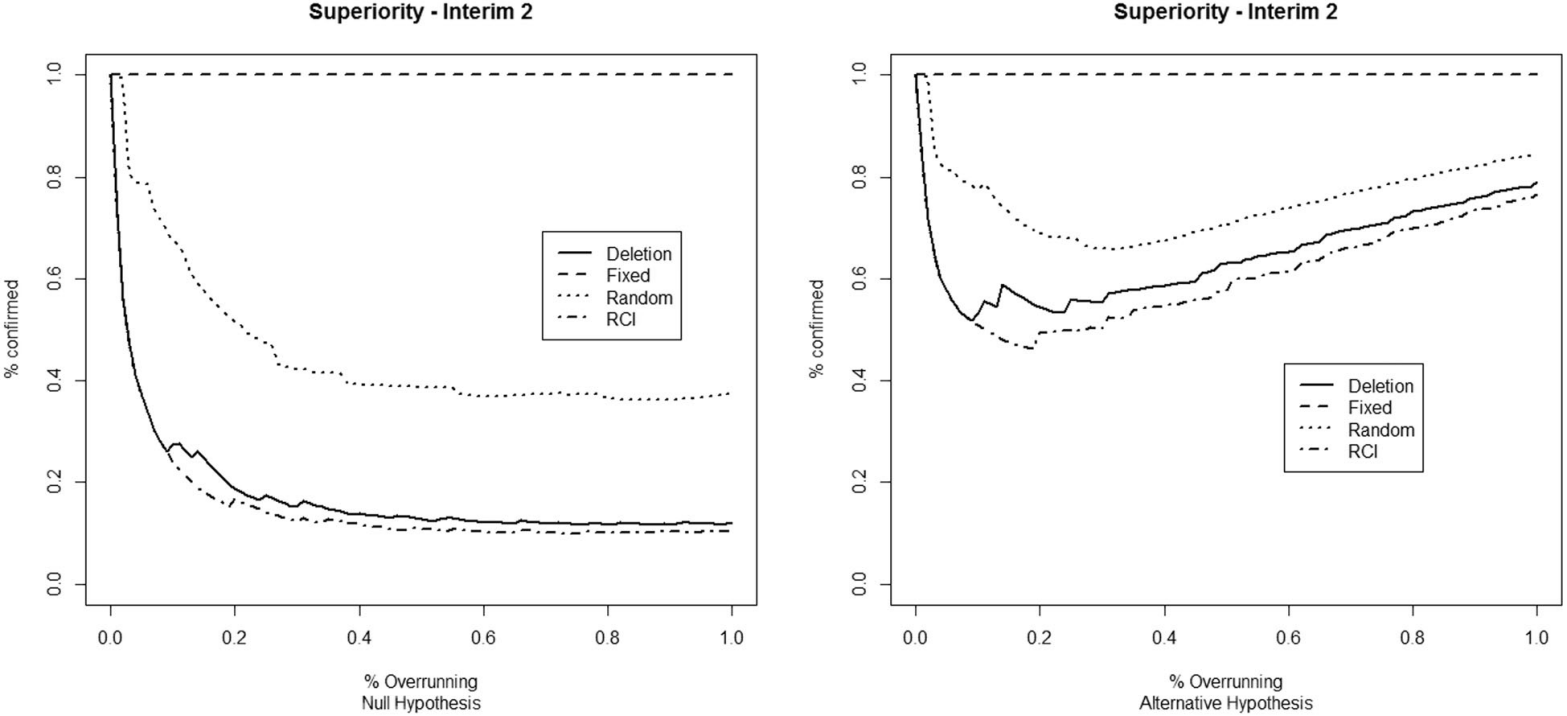
Percentage of confirming the study conclusions by the different overrunning methods at the second interim analysis. The considered trial refers to the minimum number of successes to obtain the *p* value that crosses the O’Brien-Fleming stopping bound. Data simulated under the null (left) and alternative (right) hypotheses of the superiority example

**Fig. 3:**
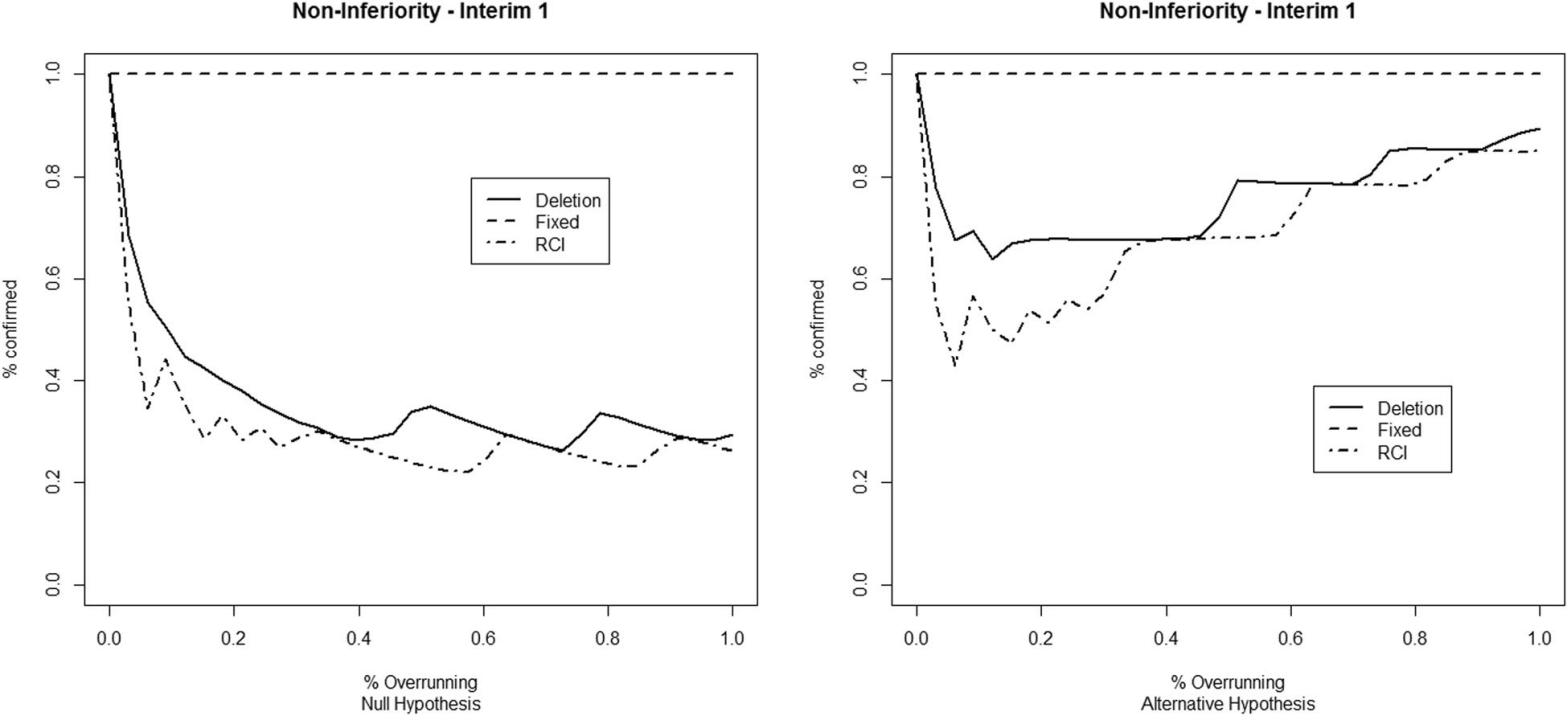
Percentage of confirming the study conclusions by the different overrunning methods at the first interim analysis. The considered trial refers to the minimum number of successes to obtain the *p* value that crosses the O’Brien-Fleming stopping bound. Data simulated under the null (left) and alternative (right) hypotheses of the non-inferiority example

**Fig. 4:**
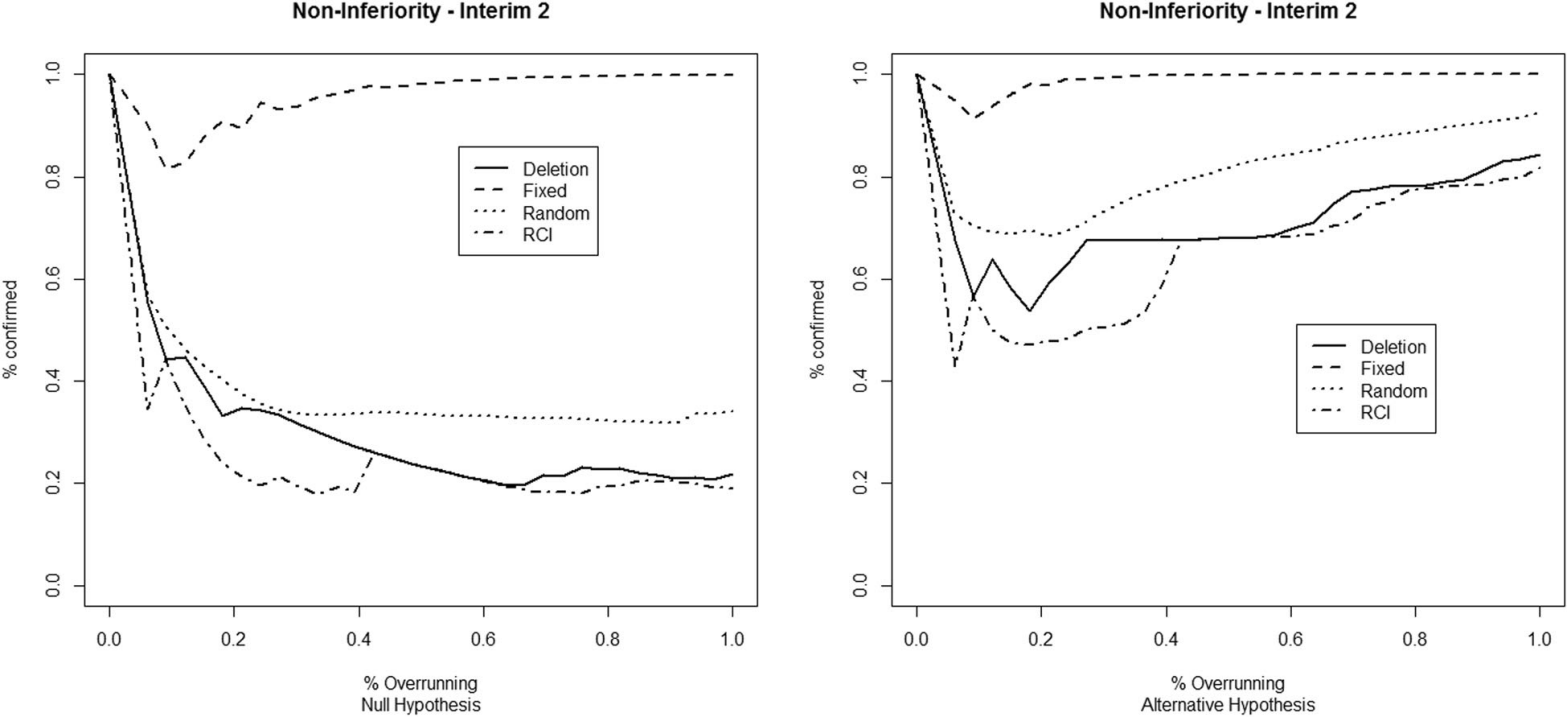
Percentage of confirming the study conclusions by the different overrunning methods at the second interim analysis. The considered trial refers to the minimum number of successes to obtain the *p* value that crosses the O’Brien-Fleming stopping bound. Data simulated under the null (left) and alternative (right) hypotheses of the non-inferiority example

The results for the first set of simulation studies are reported in Fig. 1 (first IA), Fig. 2 (second IA), and Supplementary material Table 1 for the superiority example, and in Fig. 3 (first IA), Fig. 4 (second IA), and Table 4 (Supplementary material) for the non-inferiority example. The results show how overrunning reduces the probability of confirming the study conclusions as in the IA. These reductions are present in both example trials and both at the first and the second IAs. Deletion and RCI methods seem the most conservative, while the combining *p* values method with fixed weights seems immune to the overrunning effects. The random weights method seems to have a behavior between the deletion and the fixed weights method. The fixed weights method seems not to be very sensitive to the choice of the value for the expected overrunning size, as reported in Table 2 (for the superiority example) and Table 5 (for the non-inferiority example) in the Supplementary material.

The results reported for non-inferiority example trials (Fig. 3 (first IA), Fig. 4 (second IA), and Table 4 in Supplementary material) show a greater variability of confirmation probabilities across different statistical methods used to handle with overrunning. The pattern of confirmation probabilities is similar in comparison with the superiority example.

The results of the second set of simulation studies for the superiority and non-inferiority example trials are reported, respectively, in Table 3 and Table 6 (Supplementary material). Deletion and RCI methods seem less robust. To ensure a 90% probability of confirming the study conclusions, deletion and RCI methods require, also for small overrunning size, more evidence (with *p* values between ten and one hundred times smaller) concerning combining *p* values methods. Fixed weights method seems the most robust, and indeed with 90% probability of confirming the study conclusions, the *p* values are ensured to cross the nominal significance levels (*α*_*k*_) of the O’Brien and Fleming GSD [7]. Also considering the second set of simulations, it is possible to assess a greater variability across *p* values needed to guarantee a 90% probability to confirm study results (Table 6) in non-inferiority examples in comparison with superiority trials.

## Discussion

The paper shows that the inclusion of overrunning data in a group sequential trial could lead to serious effects on the validity of early stopping reducing the probability of confirming the study conclusions in the IA. The European Medicine Agency (EMEA) guidance, in this regard, assessed that if a GSD IA result suggests an early stopping trial scenario, it could be useful to carry out an additional analysis including all the overrunning data. It may happen that when the IA is conducted, the null hypothesis could no longer be rejected, and the trial decision-making may depend on whether the overrunning patients are included in the final analysis. In such a situation, it is an accepted regulatory practice to include the overrunning data in the analysis following the intention to treat (ITT) principle [15].

In Sooriyarachchi et al. [19], a comparison between deletion and combining *p* values methods concluded that when the O’Brien and Fleming design [6] is adopted, they are in closer agreement with each other. Our simulation studies showed that the deletion method, similarly to the RCI method, is more conservative than the methods based on combining *p* values.

Considering the non-inferiority clinical trial design, international guidelines suggest the appropriateness of reporting trial results in terms of the confidence interval [26] to account also for variability in treatment effect estimation [27]. For these reasons, an RCI approach may be a suitable method to deal with overrunning data in a non-inferiority trial design setting, although RCI method presents an advantage, if compared to the other alternatives, that lies in the flexibility to hypothesis changes that do not affect the confidence interval bounds. This means that the same set of RCIs can be applied to several study hypotheses, e.g., switching from superiority to non-inferiority hypotheses [28]. Under such a scenario, the RCI method could be used, as a support tool, if the main hypotheses of the study are not so clear and switching from superiority to non-inferiority was already contemplated. In general, however, it has to be pointed out that the overrunning implies a break in the blinding, with several consequences in terms of trial analysis and reliability.

In the current literature, poor indications have been reported to deal with overrunning in non-inferiority study design; however, we dispose of indications on ITT analysis and per-protocol in non-inferiority studies [29, 30]. An ITT analysis is widely recognized as the most valid approach for superiority trials. For non-inferiority trials, instead, the inclusion of data after study drug discontinuation (ITT analysis) tends to bias the results toward equivalence. The per-protocol analysis, on the other side, excludes data from patients with evident protocol deviations potentially resulting in a bias in either direction. Therefore, non-inferiority may be assessed only if the hypothesis is supported by both ITT and per-protocol analysis [31]. Following the same line of thought, non-inferiority could be evaluated, in case of overrunning data, considering both the RCI method and the exclusion of the overrunning data from the IA. The treatment under evaluation would be considered not inferior if both analyses give consistent results.

In this sense, the simulation study shows that the effect of a small amount (about 5–10%) of overrunning data could lower the probability of confirming the study conclusions down, to below 60%. In general, it might be argued that from a clinical trial point of view, being conservative would be a good property for a method. Nevertheless, both deletion and RCI methods proved to be very conservative also when the data were simulated under the alternative hypothesis; this leads us to say that they are overly conservative. This overly conservative property is confirmed also by the requested *p* values to ensure a good probability of confirmation of the study conclusions are in the same case hundreds of times smaller than with respect to the *p* values planned by the O’Brien and Fleming GSD.

Combining *p* value with fixed weights method seems to be, on the other hand, extremely robust but also not very conservative. This method never changes the study conclusions, even in the case of a large quantity of overrunning data simulated under the null hypothesis. The method of combining *p* value with random weights placed between the conservativity of deletion and the robustness of the fixed weights methods.

A low probability of confirming the study conclusions could be due to an early stopping which implies that an extreme result was already observed, and including data not extreme as the observed, the sequential part could only worsen the results; this aspect was already underlined in Sooriyarachchi et al. [19]. Thus, this may explain why we observed a reduction of the type I error (under the null hypothesis) and the power (under the alternative) for small overrunning data portions, and it can be a possible reason that could lead the methods to regain in terms of power when the overrunning data sizes become larger.

However, the results do not allow us to suggest what is the best method. The choice should be oriented by different criteria such as the endpoint type (for safety rather than efficacy) or the design type (superiority rather than non-inferiority), in which a more or a less conservative method could be considered more suitable depending on the study design purposes.

### Limitations and further research development

The equivalence trial design has been not considered within the simulation scenarios. In several cases, non-inferiority and equivalence trials are often used interchangeably in the literature to refer to a trial design in which the primary outcome is to evaluate whatever a new treatment effect is similar to the standard comparator [32]. However, the EMEA in the International Conference on Harmonisation (ICH) clarifies the differences between the two study designs [16]. An equivalence trial may be assimilated to a two-sided test; it is a study designed to show that two interventions do not differ within a pre-specified delta margin. Instead, a non-inferiority trial is similar to a one-sided test designed to demonstrate that a new treatment is no less effective than a certain delta margin from the standard intervention [16].

The pattern of the probability of confirming the study conclusion for an equivalence trial, across different overrunning methods, is expected to correspond to non-inferiority study design with a level of significance halved. In this research article, the overrunning data are simulated close to the O’Brien and Fleming rejection boundaries. For this reason, for the equivalence trial in comparison to non-inferiority study design, less variability in the probabilities of confirming study results is expected across the different overrunning methods due to a halved significance level. Across the overrunning methods, an RCI approach may be a suitable technique to deal with overrunning data in superiority, equivalence, and non-inferiority trial design. The RCI method presents an advantage that lies in the flexibility to hypothesis changes that do not affect the confidence interval bounds. This means that the same set of RCIs can be applied to several study hypotheses, e.g., switching from superiority to non-inferiority or equivalence hypotheses [28]. Other research developments may be needed to investigate the overrunning effects on the clinical trial results according to different sizes of non-inferiority (or equivalence) margins.

The example trials considered for the simulation study are both based on the binary endpoint assessment which is a widely used outcome in the clinical research especially in phase II clinical trials [33].

The standard approaches in GSD consist of a chi-square test for binary data, a *t* test for continuous outcomes, and a log-rank test for time-to-event data. All these test statistics are approximately standard normally distributed under the corresponding null hypothesis [34]. In the literature, it has been demonstrated that the continuous outcome trials had a significantly higher power (i.e., a lower type II error rate) in comparison with the dichotomous outcome trial (or time-to-event endpoints), especially for smaller sample sizes (< 50) [35]. For this reason, concerning the impact of the overrunning methods on the clinical trial results, it is expected greater robustness of trial conclusion concerning overrunning data with a lower variability across the methods for the continuous endpoint in comparison with binary or time-to-event outcomes. Other research efforts are needed to quantify the overrunning effect on the trial conclusion with respect to the different outcome types. However, it can be assumed that the more conservative methods are well suited for non-inferiority studies and for binary endpoints due to the greater variability of clinical trial results across overrunning methods.

## Conclusions

This study evidenced that the inclusion of overrunning data could seriously change the decision of an early conclusion of the trial. Some of the methods proposed in the literature to include overrunning data are demonstrated more conservative than others.

The choice of the most suitable method to handle with overrunning data could be oriented by different criteria related to the endpoint type or the design type.

## Supplementary information

**Additional file 1: Table 1.** Confirming study conclusion rates of the overrunning methods at the first and the second IAs for some proportions of overrunning data. Results based on the simulation study for the superiority trial example. **Table 2.** Confirming study conclusion rates of fixed weights method for different choices of the expected overrunning size (rows) and the effective overruning observed (columns) in the superiority example. **Table 3.** Minimum p-values request to the overrunning methods to ensure a 90% probability of confirming study conclusions at the first and the second IAs for the superiority example. **Table 4.** Confirming study conclusion rates of the overrunning methods at the first and the second IAs for some proportions of overrunning data. Results based on the simulation study for the non-inferiority trial example. **Table 5.** Confirming study conclusion rates of fixed weights method for different choices of the expected overrunning size (rows) and the effective overruning observed (columns) in the non-inferiority example. **Table 6.** Minimum p-values request to the overrunning methods to ensure a 90% probability of confirming study conclusions at the first and the second IAs for the non-inferiority example.

## Data Availability

Please contact the author for data requests.

## Abbreviations

GSD: Group sequential design
IA: Interim analysis
RCI: Repeated confidence interval
ITT: Intention to treat
