# Supplementary material for: Overrunning in clinical trials: some thoughts from a methodological review
Source: Trials. 2020 Jul 21;21:668. doi: 10.1186/s13063-020-04526-5 (PMC7374901; doi:10.1186/s13063-020-04526-5)
Supplement: Supplementary file 1 — Additional file 1: Table 1. Confirming study conclusion rates of the overrunning methods at the first and the second IAs for some proportions of overrunning data. Results based on the simulation study for the superiority trial example. Table 2. Confirming study conclusion rates of fixed weights method for different choices of the expected overrunning size (rows) and the effective overruning observed (columns) in the superiority example. Table 3. Minimum p-values request to the overrunning methods to ensure a 90% probability of confirming study conclusions at the first and the second IAs for the superiority example. Table 4. Confirming study conclusion rates of the overrunning methods at the first and the second IAs for some proportions of overrunning data. Results based on the simulation study for the non-inferiority trial example. Table 5. Confirming study conclusion rates of fixed weights method for different choices of the expected overrunning size (rows) and the effective overruning observed (columns) in the non-inferiority example. Table 6. Minimum p-values request to the overrunning methods to ensure a 90% probability of confirming study conclusions at the first and the second IAs for the non-inferiority example. [file 13063_2020_4526_MOESM1_ESM.docx]

*Table 1 Confirming study conclusion rates of the overrunning methods at the first and the second IAs for some proportions of overrunning data. Results based on the simulation study for the superiority trial example.*

|  | **First interim** | | | **Second Interim** | | | |
| --- | --- | --- | --- | --- | --- | --- | --- |
|  | **Deletion** | **Fixed W.** | **RCI** | **Deletion** | **Fixed W.** | **Random W.** | **RCI** |
| **OR sample in favour of H_0_** |  |  |  |  |  |  |  |
| **5%** | 52.65 | 100.00 | 36.95 | 36.95 | 100.00 | 78.38 | 36.95 |
| **10%** | 34.56 | 100.00 | 25.66 | 27.45 | 100.00 | 67.54 | 24.10 |
| **15%** | 32.52 | 100.00 | 24.58 | 24.70 | 100.00 | 57.64 | 18.09 |
| **20%** | 25.52 | 100.00 | 18.68 | 18.75 | 100.00 | 51.59 | 16.66 |
| **25%** | 23.79 | 100.00 | 18.37 | 17.47 | 100.00 | 47.36 | 14.11 |
| **50%** | 17.85 | 99.99 | 14.29 | 12.77 | 100.00 | 38.46 | 10.74 |
| **75%** | 17.31 | 99.99 | 14.23 | 11.73 | 100.00 | 37.25 | 9.89 |
| **OR sample in favour of H_1_** |  |  |  |  |  |  |  |
| **5%** | 73.00 | 100.00 | 57.42 | 57.42 | 100.00 | 81.39 | 57.42 |
| **10%** | 64.38 | 100.00 | 54.32 | 53.03 | 100.00 | 77.56 | 50.80 |
| **15%** | 67.80 | 100.00 | 58.00 | 58.03 | 100.00 | 72.95 | 47.46 |
| **20%** | 63.62 | 100.00 | 54.49 | 54.49 | 100.00 | 68.93 | 49.38 |
| **25%** | 66.67 | 100.00 | 59.48 | 55.93 | 100.00 | 67.90 | 49.90 |
| **50%** | 72.20 | 100.00 | 66.91 | 62.97 | 100.00 | 70.62 | 57.64 |
| **75%** | 80.32 | 100.00 | 77.10 | 70.83 | 100.00 | 78.22 | 67.98 |

*Table 2 Confirming study conclusion rates of fixed weights method for different choices of the expected overrunning size (rows) and the effective overruning observed (columns) in the superiority example.*

|  | **OR sample in favour of H_0_** | | | | | | **OR sample in favour of H_1_** | | | | | |
| --- | --- | --- | --- | --- | --- | --- | --- | --- | --- | --- | --- | --- |
|  | **5%** | **10%** | **15%** | **20%** | **25%** | **50%** | **5%** | **10%** | **15%** | **20%** | **25%** | **50%** |
| **First Interim** |  |  |  |  |  |  |  |  |  |  |  |  |
| **Fixed 5%** | 100.00 | 100.00 | 100.00 | 100.00 | 100.00 | 100.00 | 100.00 | 100.00 | 100.00 | 100.00 | 100.00 | 100.00 |
| **Fixed 10%** | 100.00 | 100.00 | 100.00 | 100.00 | 100.00 | 99.99 | 100.00 | 100.00 | 100.00 | 100.00 | 100.00 | 100.00 |
| **Fixed 15%** | 100.00 | 99.96 | 99.90 | 99.87 | 99.85 | 99.81 | 100.00 | 100.00 | 100.00 | 99.99 | 99.99 | 100.00 |
| **Fixed 20%** | 99.83 | 99.26 | 99.20 | 99.21 | 99.22 | 99.15 | 99.98 | 99.89 | 99.87 | 99.94 | 99.94 | 99.99 |
| **Fixed 25%** | 98.97 | 97.58 | 97.37 | 97.55 | 97.72 | 97.77 | 99.81 | 99.49 | 99.55 | 99.70 | 99.80 | 99.95 |
| **Fixed 50%** | 77.96 | 83.36 | 84.05 | 84.55 | 85.02 | 85.27 | 85.92 | 93.50 | 95.28 | 96.58 | 97.44 | 99.07 |
| **Second Interim** |  |  |  |  |  |  |  |  |  |  |  |  |
| **Fixed 5%** | 100.00 | 100.00 | 100.00 | 100.00 | 100.00 | 100.00 | 100.00 | 100.00 | 100.00 | 100.00 | 100.00 | 100.00 |
| **Fixed 10%** | 100.00 | 100.00 | 100.00 | 100.00 | 100.00 | 100.00 | 100.00 | 100.00 | 100.00 | 100.00 | 100.00 | 100.00 |
| **Fixed 15%** | 100.00 | 100.00 | 100.00 | 100.00 | 100.00 | 100.00 | 100.00 | 100.00 | 100.00 | 100.00 | 100.00 | 100.00 |
| **Fixed 20%** | 100.00 | 100.00 | 100.00 | 100.00 | 100.00 | 100.00 | 100.00 | 100.00 | 100.00 | 100.00 | 100.00 | 100.00 |
| **Fixed 25%** | 100.00 | 100.00 | 100.00 | 100.00 | 100.00 | 100.00 | 100.00 | 100.00 | 100.00 | 100.00 | 100.00 | 100.00 |
| **Fixed 50%** | 74.43 | 100.00 | 100.00 | 100.00 | 100.00 | 100.00 | 73.68 | 100.00 | 100.00 | 100.00 | 100.00 | 100.00 |

*Table 3 Minimum p-values request to the overrunning methods to ensure a 90% probability of confirming study conclusions at the first and the second IAs for the superiority example.*

|  | **% Overrunning** | | | | | | |
| --- | --- | --- | --- | --- | --- | --- | --- |
|  | **5%** | **10%** | **15%** | **20%** | **25%** | **50%** | **75%** |
| **First Interim** |  |  |  |  |  |  |  |
| **Deletion** | 0.0000399 | 0.0000128 | 0.0000128 | 0.0000128 | 0.00000721 | 0.00000721 | 0.0000226 |
| **Fixed W.** | 0.000125 | 0.000125 | 0.000125 | 0.000125 | 0.000125 | 0.00022 | 0.00022 |
| **RCI** | 0.0000226 | 0.00000721 | 0.00000721 | 0.00000721 | 0.00000406 | 0.00000406 | 0.0000128 |
| **Second Interim** |  |  |  |  |  |  |  |
| **Deletion** | 0.000944 | 0.000647 | 0.000479 | 0.000479 | 0.000384 | 0.000384 | 0.000479 |
| **Fixed W.** | 0.004071 | 0.004071 | 0.004071 | 0.004071 | 0.004071 | 0.004071 | 0.004071 |
| **Random W.** | 0.002404 | 0.002404 | 0.00147 | 0.000944 | 0.000944 | 0.000647 | 0.000647 |
| **RCI** | 0.000944 | 0.000479 | 0.000479 | 0.000384 | 0.000384 | 0.00033 | 0.000384 |

*Table 4 Confirming study conclusion rates of the overrunning methods at the first and the second IAs for some proportions of overrunning data. Results based on the simulation study for the non-inferiority trial example.*

|  | **First interim** | | | **Second Interim** | | | |
| --- | --- | --- | --- | --- | --- | --- | --- |
|  | **Deletion** | **Fixed W.** | **RCI** | **Deletion** | **Fixed W.** | **Random W.** | **RCI** |
| **OR sample in favour of H_0_** |  |  |  |  |  |  |  |
| **5%** | 55.21 | 100.00 | 34.54 | 55.21 | 100.00 | 56.97 | 34.54 |
| **10%** | 50.47 | 100.00 | 44.25 | 44.25 | 90.09 | 50.70 | 44.25 |
| **15%** | 42.49 | 100.00 | 28.55 | 38.93 | 82.68 | 42.87 | 28.55 |
| **20%** | 39.91 | 100.00 | 33.21 | 34.79 | 90.93 | 37.46 | 21.41 |
| **25%** | 38.04 | 100.00 | 28.20 | 34.30 | 89.31 | 35.53 | 19.70 |
| **50%** | 34.85 | 100.00 | 23.00 | 22.93 | 97.97 | 33.45 | 22.93 |
| **75%** | 29.55 | 100.00 | 25.05 | 23.07 | 99.63 | 32.50 | 18.03 |
| **OR sample in favour of H_1_** |  |  |  |  |  |  |  |
| **5%** | 67.52 | 100.00 | 42.79 | 67.52 | 100.00 | 72.62 | 42.79 |
| **10%** | 69.16 | 100.00 | 56.61 | 56.61 | 95.03 | 70.27 | 56.61 |
| **15%** | 66.82 | 100.00 | 47.39 | 57.99 | 93.78 | 68.91 | 47.39 |
| **20%** | 67.53 | 100.00 | 53.69 | 59.25 | 98.09 | 68.37 | 47.74 |
| **25%** | 67.71 | 100.00 | 51.27 | 63.05 | 97.86 | 69.45 | 48.51 |
| **50%** | 79.13 | 100.00 | 67.92 | 67.92 | 99.92 | 82.23 | 67.92 |
| **75%** | 84.97 | 100.00 | 78.24 | 78.10 | 99.99 | 88.03 | 75.30 |

*Table 5 Confirming study conclusion rates of fixed weights method for different choices of the expected overrunning size (rows) and the effective overruning observed (columns) in the non-inferiority example.*

|  | **OR sample in favour of H_0_** | | | | | | **OR sample in favour of H_1_** | | | | | |
| --- | --- | --- | --- | --- | --- | --- | --- | --- | --- | --- | --- | --- |
|  | **5%** | **10%** | **15%** | **20%** | **25%** | **50%** | **5%** | **10%** | **15%** | **20%** | **25%** | **50%** |
| **First Interim** |  |  |  |  |  |  |  |  |  |  |  |  |
| **Fixed 5%** | 100.00 | 100.00 | 100.00 | 100.00 | 100.00 | 100.00 | 100.00 | 100.00 | 100.00 | 100.00 | 100.00 | 100.00 |
| **Fixed 10%** | 100.00 | 100.00 | 100.00 | 100.00 | 100.00 | 100.00 | 100.00 | 100.00 | 100.00 | 100.00 | 100.00 | 100.00 |
| **Fixed 15%** | 100.00 | 100.00 | 100.00 | 100.00 | 100.00 | 99.95 | 100.00 | 100.00 | 100.00 | 100.00 | 100.00 | 100.00 |
| **Fixed 20%** | 100.00 | 100.00 | 100.00 | 100.00 | 100.00 | 99.74 | 100.00 | 100.00 | 100.00 | 100.00 | 100.00 | 100.00 |
| **Fixed 25%** | 100.00 | 100.00 | 100.00 | 99.68 | 99.17 | 98.76 | 100.00 | 100.00 | 100.00 | 99.95 | 99.89 | 99.98 |
| **Fixed 50%** | 100.00 | 90.09 | 85.54 | 86.97 | 88.13 | 88.00 | 100.00 | 95.03 | 96.31 | 97.53 | 98.10 | 99.49 |
| **Second Interim** |  |  |  |  |  |  |  |  |  |  |  |  |
| **Fixed 5%** | 100.00 | 100.00 | 98.99 | 98.94 | 99.57 | 99.95 | 100.00 | 100.00 | 99.76 | 99.82 | 99.96 | 100.00 |
| **Fixed 10%** | 100.00 | 90.09 | 82.68 | 90.93 | 89.31 | 97.97 | 100.00 | 95.03 | 93.78 | 98.09 | 97.86 | 99.92 |
| **Fixed 15%** | 100.00 | 56.97 | 74.39 | 79.99 | 81.03 | 93.10 | 100.00 | 72.62 | 88.83 | 94.35 | 95.50 | 99.54 |
| **Fixed 20%** | 100.00 | 56.97 | 66.93 | 79.99 | 77.27 | 89.38 | 100.00 | 72.62 | 85.91 | 94.35 | 93.57 | 99.33 |
| **Fixed 25%** | 100.00 | 56.97 | 66.93 | 68.29 | 76.62 | 85.22 | 100.00 | 72.62 | 85.91 | 89.30 | 93.48 | 98.70 |
| **Fixed 50%** | 100.00 | 56.97 | 46.20 | 53.36 | 58.82 | 65.69 | 100.00 | 72.62 | 69.07 | 81.05 | 83.91 | 94.55 |

*Table 6 Minimum p-values request to the overrunning methods to ensure a 90% probability of confirming study conclusions at the first and the second IAs for the non-inferiority example*

|  | **% Overrunning** | | | | | | |
| --- | --- | --- | --- | --- | --- | --- | --- |
|  | **5%** | **10%** | **15%** | **20%** | **25%** | **50%** | **75%** |
| **First Interim** |  |  |  |  |  |  |  |
| **Deletion** | 0.0000182 | 0.0000182 | 0.0000182 | 0.0000182 | 0.0000182 | 0.0000182 | 0.0000534 |
| **Fixed W.** | 0.000155 | 0.000155 | 0.000155 | 0.000155 | 0.000155 | 0.000155 | 0.000155 |
| **RCI** | 0.0000182 | 0.0000182 | 0.00000608 | 0.00000608 | 0.00000608 | 0.00000608 | 0.0000182 |
| **Second Interim** |  |  |  |  |  |  |  |
| **Deletion** | 0.000703 | 0.000703 | 0.000414 | 0.000414 | 0.000414 | 0.000414 | 0.000703 |
| **Fixed W.** | 0.003829 | 0.003829 | 0.003829 | 0.003829 | 0.003829 | 0.003829 | 0.003829 |
| **Random W.** | 0.001519 | 0.001519 | 0.000703 | 0.000703 | 0.000703 | 0.001519 | 0.001519 |
| **RCI** | 0.000259 | 0.000259 | 0.000259 | 0.000259 | 0.000259 | 0.000259 | 0.000259 |
